# An Evaluation of Potential Occupational Exposure to Asbestiform Amphiboles near a Former Vermiculite Mine

**DOI:** 10.1155/2009/189509

**Published:** 2009-11-23

**Authors:** Julie F. Hart, Terry M. Spear, Tony J. Ward, Caitlan E. Baldwin, Marissa N. Salo, Mohamed I. Elashheb

**Affiliations:** ^1^Department of Safety, Health, and Industrial Hygiene, Montana Tech of The University of Montana, 1300 W. Park Street, Butte, MT 59701, USA; ^2^Center for Environmental Health Sciences, The University of Montana, Missoula, MT 59812, USA

## Abstract

Amphibole asbestos (AA) has been detected on the surface of tree bark in forests neighboring an abandoned vermiculite mine near Libby, Montana. In the present study, simulations were performed to assess potential AA exposure associated with United States Department of Agriculture Forest Service (FS) occupational activities. Bark samples were collected prior, and personal breathing zone (PBZ) and Tyvek clothing wipe samples were collected during and immediately after trials that simulated FS activities. Transmission electron microscopy (TEM) analyses revealed AA bark concentrations up to 15 million structures per square centimeter (s/cm^2^). AA was detected in 25% of the PBZ TEM samples. AA was detected on wipe samples collected from all activities evaluated. This research demonstrates the potential for airborne exposure and transport of AA in the Kootenai National Forest. These findings are especially relevant to those that work in the area and to the general public who may conduct recreational activities.

## 1. Introduction

Libby, Montana (population ~2700, with nearly 12 000 in the surrounding area) is located in northwest Montana and was once home to one of the world's largest vermiculite mines. While the Libby vermiculite had useful insulating and soil conditioning properties, ore from the mine (in operation from the 1920s–1990) was contaminated with fibrous and nonasbestiform amphiboles in veins throughout the deposit [1]. Approximately 30–40% of the amphiboles are asbestiform and include winchite, richterite, tremolite, and magnesioriebeckite; differing in their relative proportions of cations (Mg, Ca, Fe, Na, K) [2–7]. 

Over 70 years of mining amphibole-contaminated vermiculite has led to amphibole asbestos (AA) contamination in areas surrounding the abandoned mine and in other areas throughout the town. Libby was added to the Environmental Protection Agency's (EPA) National Priorities List in October 2002. In 2005, researchers discovered that trees surrounding the former vermiculite mine served as reservoirs for AA [8]. Transmission electron microscopy (TEM) analysis of bark samples from trees near the vermiculite mine yielded amphibole fiber concentrations in excess of 100 million amphibole structures per square centimeter of bark surface (s/cm^2^). Contamination has also been identified in trees near transportation corridors where vermiculite was transported from Libby to processing facilities around the country [8].

In 2006, research was conducted to assess potential exposure to AA associated with harvesting firewood within the EPA-restricted zone [9]. Personal breathing zone (PBZ) and Tyvek clothing wipe samples revealed that AA was liberated from tree bark during harvesting tasks and that a potential exists for direct inhalation exposure and clothing contamination.

In September, 2007, EPA and W.R. Grace entered into an agreement to determine the nature and extent of contamination and any threat to the public health, welfare, and the environment caused by the release or threatened release of hazardous substances, pollutants, or contaminants at or from the former mine site. In 2007/2008 EPA contractors collected bark samples from forested areas surrounding the former mine site and found AA bark contamination ranging from less than the limit of detection (LOD) to 20 million s/cm^2^. AA contamination on tree bark extends several kilometers (km) from the mine site outside of the EPA restricted zone [10].

Occupational exposure to AA is associated with significant increases in asbestosis, lung cancer, and pleural cancer compared to the rest of the U.S. population [11]. High incidences of asbestos-related disease have been reported in former mine and mill workers [12–14]. While asbestos-related disease in the general Libby population has also been reported, risk associated with lower level exposures has not been as clearly defined. Medical testing of persons who lived or worked in the Libby area for at least six months before 1991 showed pleural abnormalities (calcifications, thickenings, or plaques) in 17.8% of 6668 participants [15]. Although the focus of the [15] study was to describe lung abnormalities in the general Libby population, significant factors for predicting pleural abnormalities included occupational pathways [16]. Additional occupational and nonoccupational mesothelioma cases have been identified since the end of the last follow-up [17–19], and current mortality figures indicate one new case per year in Lincoln County, Montana. For the last five-year period for which figures are available (2000–2004), there were five mesothelioma deaths (two female) in Lincoln County, making it the third-highest county in the USA in terms of age-adjusted death rate per million population at 56.1 [20].

 Much of the land surrounding the former vermiculite mine is owned by the United States Department of Agriculture (USDA) and private logging companies. USDA Forest Service (FS) personnel frequently travel on roadways and trails in the Kootenai forest. To date, there have been no occupational exposure assessments of FS employees pertaining to AA. The purpose of this research was to evaluate the potential for occupational AA exposure as a result of FS activities in the Kootenai National Forest. The potential for AA exposure was evaluated through the analysis of PBZ samples and Tyvek clothing wipe samples collected during and immediately after trials that simulate FS tasks.

## 2. Material and Methods

### 2.1. Preliminary Work

Preliminary work for this research was conducted in the fall of 2007. Investigators met with FS personnel and discussed tasks typically performed (and roadways and trail systems most commonly used) in areas within an 8 km radius of the former vermiculite mine. In addition, prevailing wind data via a Windrose were obtained [21].

Tree bark samples were also collected during this time to determine if AA contamination was present in areas frequented by FS personnel near the former vermiculite mine, but outside of the EPA's restricted zone, and within prevailing wind locations from the mine. Bark samples were collected from several tree species: Tamarack (Larix laricina), Douglas fir (Pseudotsuga menziesii), and Ponderosa pine (Pinus ponderosa) employing [8] methods. The location of each tree sampled was identified and recorded using a Garmen Etrex 12 channel global positioning system (GPS). A minimum of one 200 gram bark sample was collected from two sides of each tree approximately 1.2 m from the base. The bark was collected by prying off sections with a small pry bar and placing them in labeled plastic bags. The bags were then sealed and the pry bar was cleaned with a wet wipe after each collection. The bark samples were preserved for later analysis by TEM.

The activities selected for evaluation included driving on roadways, walking through forested areas, performing tree measurement activities, performing trail maintenance, and constructing a fire line. Tree measurement, trail maintenance, and fire line construction activities were demonstrated by FS personnel in an area with no known AA contamination. Tree measurement tasks are typically performed by at least two foresters in a plot of 10–12 trees. Tree diameter is measured with diameter tape. Tree height is then measured by securing loggers tape to the tree surface approximately 1.2 m from the ground and walking while unrolling the tape 9–15 m away from the tree. A clinometer is then used to indirectly measure tree height. Along with tree diameter and tree height, tree measurement activities usually include visually evaluating all the trees in the plot for disease. 

Fire line construction is performed by a minimum of four foresters. The objective of the fire line is to construct a 1-2 m fuel break with combustible materials cleared to a mineral soil base. The type of fire line constructed, flat scrape or cup trench, is dependent on the slope grade. The first task performed in fire line construction is removal of trees and brush. This is performed by a chainsaw operator and a brush clearer. A Pulaski tool, a comby (combination) tool, and/or a Rogue hoe are then used to clear vegetation approximately 30–35 cm to mineral soil.

Trail maintenance activities are similar to fire line construction in that a chainsaw operator and brush clearer remove vegetation growth from the trail; however, the trail is not cleared to mineral grade soil. Trail maintenance also involves a wider corridor 2-3 m, and trees are limbed with the chainsaw to a height of 2.4 m to allow for transportation by horseback.

FS personnel do not currently employ the use of personal protective equipment (PPE) beyond level D when performing field tasks in the Kootenai forest. Therefore, the tasks typically conducted by FS personnel were simulated by the research team. In an effort to minimize risks associated with the task simulations, FS personnel provided training on vehicle safety procedures, emergency radio communication, procedures for minimizing hunting related risks, and procedures for wild animal encounters. The investigators were also issued a radio for emergency communication. The investigators were suited in level C PPE while performing task simulations. This PPE consisted of hooded Tyvek coveralls, neoprene gloves, Tyvek booties, a half mask air purifying respirator with P100 filters, work boots, hard hat, and orange reflective vests (during hunting season only). All investigators obtained medical clearance to wear negative pressure respiratory protection and passed quantitative fit tests within the past year. This project was approved by the University of Montana's Institutional Review Board for the Use of Human Subjects in Research.

The PPE selected for this research presented a potential heat stress risk to the investigators. This risk was minimized by conducting the task simulations in the early morning and evening hours. In addition, task durations associated with the most physical simulation, fire line construction, were minimized and adequate fluid intake and work breaks were emphasized.

### 2.2. Research Methods

Simulations were performed in July of 2008. The meteorological conditions during the sampling period included temperatures from 15.8 to 25.5 °C, 20%–24% humidity, and wind speeds from 8–18 km per hour. Morning dew condensation on vegetation was observed during early morning trials, but no measured precipitation was reported.

**Figure 1:**
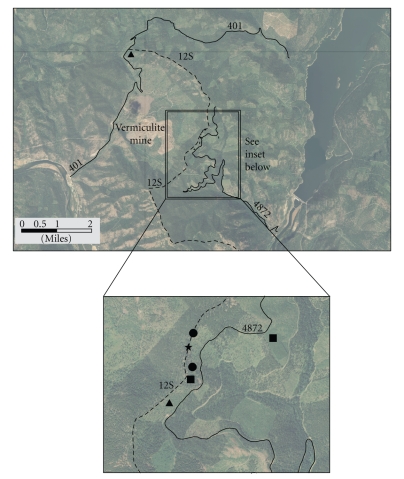
Map illustrating the location of Forest Service activity task simulations in relation to the former vermiculite mine. United States Department of Agriculture Forest Service Roadways 4872 and 401 and Rainy Divide Trail/Loop 12S are identified. Simulation activities are identified via the following symbols: fire line construction: triangle; tree measurement: square; walking: circle; and trail maintenance: star.

Two simulation trials each were performed for the following tasks: (1) driving on FS roads, (2) walking through forested areas, (3) tree measurement, and (4) fire line construction activities. In addition, one trail maintenance activity was performed. One driving simulation was also conducted in November of 2007, when preliminary data collection necessitated roadway driving. All of the simulations were conducted on FS land north and east of the former mine and EPA-restricted zone (Figure 1). 

Potential AA exposure was assessed via PBZ sampling and Tyvek clothing wipe sampling for all tasks with the exception of roadway driving. The roadways selected for the roadway driving task include FS Roads 4872 and 401 (Figure 1). Prior to driving up these roadways from paved access ways, a 10 × 10 cm disposable Manila template was secured to the rear vehicle bumper with duct tape. The template was then wiped three times with SKC Ghost wipes premoistened with deionized water. These wipes were then discarded and a 4th wipe was used to gather a pretravel vehicle wipe. The wipe sampling protocol followed the American Society for Testing and Materials (ASTM) D 6480-05 procedures, Wipe Sampling for Settled Asbestos [22]. This 4th wipe was placed in a labeled plastic bag and sealed. The vehicle was then driven to the terminal destination (Figure 1) and parked while the investigators got out of the vehicle and performed other task simulations. Other task simulations were conducted at least eight meters from the vehicle. Investigators then returned to the vehicle and drove down the roadways to the same location where the pretravel vehicle wipe was collected. A posttravel vehicle wipe sample, employing the methods described above, was then collected and placed in a labeled plastic bag and sealed. The wipe samples were analyzed for asbestos per ASTM's D 6480-05 Method, TEM Asbestos Analysis [22] by ALS Laboratories (Cincinnati, OH), a laboratory accredited by the American Industrial Hygiene Association (AIHA) (PCM), the National Voluntary Laboratory Accreditation Program (NVLAP) (TEM), and the New York State Department of Health Environmental Laboratory Approval Program (PCM and TEM). Wipe samples submitted included ten percent field blanks.

The total distances driven for the FS Roadway 4872 and 401 activities were 25 and 21 km, respectively. The average vehicle speed was 16–24 km per hour. Other vehicle traffic, ahead of the test vehicle, was noted for the November roadway driving assessment, and no other vehicle traffic was observed during the remaining roadway driving activities. 

PBZ samples were collected during the walking, tree measurement, fire line construction, and trail maintenance simulation trials using conductive three piece asbestos sampling cassettes. The cassettes contained 25 mm 0.8 micron (*μ*m) pore size mixed cellulose ester membrane filters. SKC Aircheck 224 sampling pumps were calibrated before and after each trial with a Bios Defender 520 primary flow meter at an average flow rate of three liters per minute (L/min). Throughout each trial, each investigator wore a sampling pump with the asbestos cassette placed in the breathing zone. PBZ samples were analyzed for fibers per National Institute for Occupational Safety and Health's Manual of Analytical Method (NMAM) 7400, Asbestos and Other Fibers by phase contrast microscopy (PCM) [23], and for asbestos per EPA's Asbestos Hazard Emergency Response Act's (AHERA), Airborne Asbestos by TEM [24]. AHERA requires selected area electron diffraction and energy dispersive X-ray analysis to determine mineral type and elemental composition (asbestos types). Fibers classified as “actinolite/tremolite” also included the winchite/richterite fibers characterized by Meeker et al. [6]. Asbestos structures greater than 0.5 *μ*m long with an aspect ratio (length : width) greater than or equal to 5 : 1 are recorded in the AHERA analysis. Data were reported as the concentration of asbestos structures less than (<) 5 *μ*m long and the concentration of asbestos structures greater than or equal to (≥) 5 *μ*m long. All air samples were analyzed by ALS Laboratories. PBZ samples submitted included ten percent field blanks.

In addition to PBZ sampling, surface wipe sampling of the outer layer of Tyvek clothing was conducted at the conclusion of each walking, tree measurement, fire line construction, and trail maintenance simulation trial. The wipe sampling protocol followed the American Society for Testing and Materials (ASTMs) D 6480-05 procedures, Wipe Sampling for Settled Asbestos [22]. Wipes were collected with SKC Ghost wipes premoistened with deionized water. A 10 by 10 cm SKC disposable Manila paper template was used for each wipe. A wipe sample was gathered on each investigator's chest, forearm, and shin. The site of the chest, forearm, and shin sample (right/left) was randomly selected. The three wipe samples collected for each investigator were submitted for analysis as a composite sample. In addition to the postsimulation trial wipes collected, presimulation trial wipes and ten percent field blanks were collected and analyzed. The wipe samples were analyzed for asbestos per ASTM's D 6480-05 Method, TEM Asbestos Analysis [22] by ALS. 

The average duration of each activity simulation was 66 minutes. The fire line construction activities were conducted for 31–42 minutes and the remaining task durations were 70–90 minutes. The fire line activity was shorter in duration simply because of the physical nature of the task. An effort was made to minimize potential overloading of the PBZ filters and, as described above, a shorter duration was selected for the fire line construction activities to minimize potential heat stress hazards to the investigators. 

FS personnel loaned the research team equipment in order to perform task simulations. The tools included a new Stihl Model MS361 chainsaw, Pulaski tool, comby tool, diameter tape, clinometer, and forester tape. These tools were wiped with wet wipes prior to and immediately after each simulation trial. At the conclusion of the fire line construction and trail maintenance trials, and prior to equipment cleaning, one wipe sample was collected on the chainsaw bar. The wipe samples were collected using the methods described above and placed in labeled bags and sealed. The wipe samples were analyzed for asbestos per ASTM's D 6480-05 Method, TEM Asbestos Analysis [22] by ALS. 

A minimum of two investigators conducted the walking simulation trials. The tree measurement simulation trials were conducted by three investigators; two investigators conducted tree diameter and height measurements, while the third investigator served as the data recorder. Fire line construction simulation trails were conducted with five investigators; one investigator each served as a chainsaw operator, brush clearer, Pulaski tool operator, comby tool operator, and data recorder. Five investigators conducted the trail maintenance simulation trials; one served as a chainsaw operator, three served as brush clearers, and one was the data recorder.

All simulation activities were performed within a 4.8 km radius of the former vermiculite mine. Fire line construction simulations were conducted near the Rainy Divide stock trail head (12S) and in a forested area northwest of FS roadway 4872. Tree measurement simulation activities were performed in the Alexander Test Site and in a forested area northwest of FS roadway 4872. Trail maintenance simulation activities were performed on the Rainy Divide Trail (12S). Walking activities were performed in the forested area northwest of FS roadway 4872 and Rainy Divide Trail (12S). The location of each simulation trial in relation to the former vermiculite mine is illustrated in Figure 1. The area selected for the majority of the simulations, near roadway 4872, is accessible by vehicle travel for approximately 8 km up roadway 4872 from the paved roadway (228) (Figure 1). Past this point, the roadway is currently restricted to general public vehicle traffic but may be accessed by nonmechanized means or FS vehicles. The Rainy Divide stock trail head (12S) is available for general public and FS travel from the northern section of roadway 401 (Figure 1).

## 3. Results

### 3.1. Tree Bark Sampling Results

Seven bark samples collected from trees northeast of the former vermiculite mine showed substantial AA contamination, ranging from 37 thousand to 15 million structures/cm^2^ of bark surface area (Table 1). These concentrations are consistent with AA contamination in tree bark previously reported by Ward et al. [8]. Fiber dimension analyses of the bark samples revealed that the majority of the asbestos fibers detected were <5 *μ*m long. Fibers exhibited mineral characteristics consistent with Libby amphiboles. Amphibole fibers were not detected in bark sample collected from the Missoula, MT tree (control).

### 3.2. PBZ Sampling Results

PBZ samples collected during the FS simulation activities were analyzed for asbestos by both PCM and by AHERA TEM. Table 2 presents individual sample and Mean PBZ air sampling results, reported for each simulation activity (fire line construction, tree measurement, trail maintenance, and walking) as well as by the task(s) associated with the activity. Mean concentrations were calculated by using a value of zero for nondetect concentrations. In terms of TEM mean concentrations, this method may reflect an uncertain estimate of true mean and actual risks may be higher or lower [25]. Fibers were observed on all samples analyzed by PCM, excluding field blanks.

The current occupational 8-hour time weighted average (TWA) exposure limit for asbestos is 0.1 fiber per mL for fibers >5 *μ*m long, with an aspect ratio greater than or equal to 3 : 1, as determined by PCM (OSHA, ACGIH, 2001). The National Institute for Occupational Safety and Health (NIOSH) recommended that exposure limit for asbestos is identical except that it is based on a 10-hour TWA (NIOSH). In addition to the TWA permissible exposure limit, OSHA has defined an excursion limit of 1.0 fiber per mL averaged over a sampling period of 30 minutes. 

For individual PBZ FS simulation trial samples for fibers >5 *μ*m, 10 of 24 samples (forty-two percent) exceeded the OSHA exposure limit of 0.1 fiber per mL, assuming an eight-hour exposure duration, when analyzed by PCM. These 10 PBZ samples were all collected during the fire line construction simulation activity.

A substantial portion of cellulose (from forest vegetation) fibers was expected in PCM analyses; therefore, AHERA TEM analyses were performed to describe the fiber population. In terms of fiber counts reported by the laboratory (not shown in Table 2), one to five nonasbestos fibers (organic, gypsum) were identified on all PBZ AHERA TEM samples. Twenty-five percent of the PBZ samples revealed concentrations greater than the analytical sensitivity (AS) when analyzed by AHERA TEM. These samples were collected during the fire line construction and tree measurement simulation activities. AHERA TEM analyses for the concentration of asbestos fibers >5 *μ*m revealed that none of the samples collected exceeded the OSHA PEL, assuming an 8-hour exposure duration (not shown in Table 2). 

Although the simulations for each task were conducted in two separate geographical areas (Figure 1), no differences in PBZ concentrations were observed for each simulation based on the area that the simulation activity was conducted (not shown in Table 2). 

The tasks that revealed PBZ concentrations greater than the AS for the fire line construction activity were brush clearer (2 of 2 samples) and Pulaski tool operator (2 of 2 samples). Two of five tree maintenance activity samples revealed concentrations greater than the AS. One of the walking activity PBZ samples revealed chrysotile asbestos (not shown in Table 2). Chrysotile asbestos is not part of the amphibole family, and this PBZ sample contamination may have been derived from sources other than the vermiculite mine. 

A review of the scanning electron microscope (SEM) energy dispersive X-ray spectroscopy (EDS) spectra (not shown) for PBZ samples with detectable amphibole asbestos revealed measurable amounts of sodium and potassium in 100% of the samples. Recent research has demonstrated that amphiboles originating from the vermiculite deposit contain sodium and potassium that can be observed in the SEM-EDS spectra [5].

### 3.3. Wipe Sampling Results

Surface wipe sampling of the outer layer of Tyvek clothing was conducted at the conclusion of each activity simulation trial. These wipe samples were analyzed for asbestos fibers by TEM, with summary results presented in Table 3. All of the field blank and preactivity Tyvek wipe samples showed no asbestos contamination and were below the AS (448 structures per cm^2^) for the D 6480-05 TEM method. Fifty-two percent of postactivity wipe samples revealed concentrations greater than the AS. While the concentrations of AA were associated with the fire line construction activity, AA was detected on wipe samples collected from all of the activities evaluated. 

The tasks that revealed wipe sample concentrations greater than the AS for the fire line construction activity were brush clearer (1 of 1 sample), comby tool operator (1 of 2 samples), and Pulaski tool operator (2 of 2 samples). Four of the five tree measurement activity samples revealed concentrations greater than the AS. Two of three trail maintenance brush clearer and one of one trail maintenance chainsaw operator samples was greater than the AS, while one of five walking samples were greater than the AS. As noted with one PBZ walking sample, one of the walking activity wipe samples (not reported) revealed chrysotile asbestos. As noted previously, chrysotile asbestos is not part of the amphibole family. However, fibers and bundles with split ends resembling commercial grade asbestos have been identified but are not common in the Rainy Creek Complex near Libby, Montana [6]. 

The pre- and post- travel vehicle wipes collected for the FS 4872 vehicle driving activity simulations revealed concentrations below the AS for both the November and July trials. The pretravel vehicle wipe collected for the FS 401 vehicle driving activity simulation was also reported below the AS, while the posttravel wipe sample revealed one amphibole fiber resulting in a concentration of 17 917 s/cm^2^. The amphibole fiber detected was less than 5 *μ*m long.

Postactivity chainsaw bar wipe sample results are presented in Table 4. AA was detected on the chainsaw bar after all of the simulation activities. In terms of structure counts reported by the laboratory, 12 of 15 fibers were less than 5 *μ*m long (not shown).

A SEM-EDS spectra (not shown) for all wipe samples (clothing, equipment, vehicle) with detectable amphibole asbestos revealed measurable amounts of sodium and potassium in 73% of the samples.

## 4. Conclusions

Results from the FS activity simulations conducted within this study indicate that an exposure to AA may exist when work is performed in the Kootenai National Forest near the former vermiculite mine. Bark samples collected in the area where activity simulations were conducted revealed amphibole contamination ranging from 37 thousand to 15 million structures per cm^2^ of bark surface area. The lowest bark amphibole concentrations were observed in the Alexander Test Site, an area that was replanted after a timber harvest in the early 1990s as a research plot for Tamarack trees. It is worth noting that trees in this location were planted after the vermiculite mine ceased operations. Contamination of these trees may indicate more recent dispersion of amphibole fibers. The highest bark amphibole concentrations were observed in aged Douglas Fir trees on the Rainy Divide Trail.

In terms of inhalation exposure potential associated with the FS tasks evaluated, fire line construction and tree measurement activities yielded detectable AS TEM PBZ concentrations. Detectable AA concentrations were not observed with trail maintenance and walking activities. Of the fire line activity tasks evaluated, the Pulaski tool operator and the brush clearer yielded the highest PBZ concentrations. PBZ concentrations for these fire line activity tasks revealed detectable AA concentrations for two separate trials conducted in two separate geographical areas. Five PBZ samples were collected for the tree maintenance activity. Of these, three samples revealed detectable AA. These three samples were also collected in two separate trials conducted in two separate geographical areas. In terms of individual fiber counts, fifty-seven percent of PBZ asbestos structures were <5 *μ*m long. This is consistent with other research performed regarding amphibole asbestos in tree bark [8, 9].

It is worth noting that the operation of the Pulaski tool employed in fire line construction involves clearing vegetation to mineral grade soil. Therefore, it is unclear whether AA exposure associated with this task is derived from vegetation or soil sources.

In addition to the airborne exposure potential associated with FS activities, there is a potential for clothing and equipment contamination. Composite wipe samples collected from the investigators′ forearm, shin, and chest revealed detectable amphibole asbestos in fifty-two percent of the samples collected. Clothing contamination was observed in samples from each of the four activities evaluated: fire line construction, tree measurement, trail maintenance, and walking. In addition, the wipe samples collected from the chainsaw bar after each trial (*n* = 3) revealed amphibole contamination ranging from 896 to 11 825 s/cm^2^. Clothing and equipment contamination may serve as a secondary source of exposure to FS personnel. Cross contamination of vehicle cabs, vehicle boxes, equipment storage areas, equipment maintenance areas, and offices may occur as a result of clothing and equipment contamination. 

Although the objective of this study was to assess the potential exposure associated with FS occupational activities, the potential for public exposure to AA cannot be ignored. Libby and the surrounding area are known for clean water, beautiful scenery, and recreational activities such as hiking, hunting boating, and skiing. As noted earlier, the simulation areas are accessible to the general public. The frequency of recreational use by the general public was not evaluated in this study; however, hunters were observed near the simulation site during the bark collection phase of this study. In an effort to inform the public about the amphibole contamination in the Kootenai National Forest, FS management has published a brochure that outlines safeguards to minimize dust generation and transport of fibers on clothing.

The forested areas near the simulation sites were historically used for timber harvests as observed by numerous clear-cut plots. In the past, FS personnel visited the Alexander Test plot and areas accessible via roadways 4872 and 401 on a weekly basis. Since the awareness of amphibole contamination in tree bark, FS travel in this area has been reduced. In addition, fire fighting in this area is currently performed from the air only.

This research was funded as a small project/pilot study in order to assess potential FS exposure to AA. A limited number of samples were collected within a relatively small geographical area. Future research is planned to assess FS exposure potentials with the activities evaluated in this study throughout a range of meteorological conditions (i.e., different seasons) as well as other activities (i.e., fire fighting), in expanded radii from the former vermiculite mine. In addition, vehicle cabs, offices, and equipment storage and maintenance facilities should be evaluated for potential AA contamination.

## 5. Competing Interests

One of the authors (TMS) has served as an expert witness for plaintiffs′ attorneys in litigation involving asbestos exposure in Libby, MT.

## Figures and Tables

**Table 1:** Tree bark sample results—Forest Service land northeast of the former vermiculite mine.

Location *n* = (8)	Tree Species	Amphibole structures/cm^2^
Alexander Test Site	Tamarack	36,898
Alexander Test Site	Tamarack	158,583
Alexander Test Site	Tamarack	112,336
Rainy Divide Trail 12S	Ponderosa pine	568,137
Rainy Divide Trail 12S	Douglas Fir	12,356,979
Rainy Divide Trail 12S	Douglas Fir	15,383,941
Rainy Divide Trail 12S	Douglas Fir	13,377,926
Bark sample collected in Missoula,	Ponderosa pine	^(a)^ND
MT. Serves as control sample		

^(a)^ND = Nondetect.

**Table 2:** PBZ data reported by activity performed, task associated with activity, PCM and TEM individual, and mean sample concentrations.

Test activity	Test activity task	Number of samples	^ (a)^Number of detects (PCM)	PCM conc. (fibers/mL)	^ (b)^Number of detects (TEM)	TEM conc. <5 *μ*m (structures/mL)	TEM conc. >5 *μ*m (structures/mL)	TEM conc. total structures (structures/mL)
Fire Line		10	10		4			

	Brush Clearer			0.354		0.0277	0.0277	0.0544
				0.249		ND	0.0367	0.0367
	Chainsaw Operator			0.384		ND	ND	ND
				0.242		ND	ND	ND
	Pulaski Operator			0.438		0.0524	0.0262	0.0786
				0.410		0.0332	0.0664	0.0996
	Comby Operator			0.446		ND	ND	ND
				0.238		ND	ND	ND
	Data Recorder			0.220		ND	ND	ND
				0.117		ND	ND	ND

	Mean Concentrations			0.302		0.011	0.016	0.027

Trail Maintenance		5	5		0			

	Brush Clearer			0.059		ND	ND	ND
				0.045		ND	ND	ND
				0.024		ND	ND	ND
	Chainsaw Operator			0.063		ND	ND	ND
	Data Recorder			0.015		ND	ND	ND

	Mean Concentrations			0.041		ND	ND	ND

Tree Measurement	Tree Measurer	5	5		2			

				0.021		ND	ND	ND
				0.035		ND	0.0162	0.0162
				0.015		ND	ND	ND
				0.038		0.0134	ND	0.0134
				0.062		ND	ND	ND

	Mean Concentrations			0.034		0.003	0.003	0.006

^ (c)^Walking	Walking	4	4		0			

				0.021		ND	ND	ND
				0.043		ND	ND	ND
				0.011		ND	ND	ND
				0.019		ND	ND	ND

	Mean Concentrations			0.024		ND	ND	ND

^(a)^PCM Analytical Limit of Detection : (0.009 − 0.19 f/mL). ^(b)^ND : Non detect, TEM analytical sensitivity : (0.0123 − 0.0367 s/mL). ^(c)^One walking activity PBZ sample (not reported) revealed chrysotile asbestos, not amphibole asbestos.

**Table 3:** Clothing wipe sample data reported by activity performed, task associated with activity, individual, and mean TEM concentrations.

Test Activity	Test Activity Task	Number Of Samples	^ (a)^Number Of Detects (TEM)	TEM Conc. <5 *μ*m (structures/cm^2^)	TEM Conc. >5 *μ*m (structures/cm^2^)	TEM Conc. Total Structures (structures/cm^2^)
Fire Line		8	4			

	Brush Clearer			896	ND	896
	Chainsaw Operator			ND	ND	ND
				ND	ND	ND
	Pulaski Operator			1,344	1,792	3,135
				448	448	896
	Comby Operator			896	ND	896
				ND	ND	ND
	Data Recorder			ND	ND	ND

	Mean Concentrations			448	280	728

Trail Maintenance		5	3			

	Brush Clearer			299	ND	299
				299	ND	299
				ND	ND	ND
	Chainsaw Operator			1,792	ND	1,792
	Data Recorder			ND	ND	ND

	Mean Concentrations			478	ND	478

Tree Measurement	Tree Measurer	5	4			

				ND	ND	ND
				ND	448	448
				448	ND	448
				ND	448	448
				448	448	896

	Mean Concentrations			179	269	448

^ (b)^Walking	Walking	5	1			

				ND	ND	ND
				ND	ND	ND
				ND	ND	ND
				896	ND	896
				ND	ND	ND

	Mean Concentrations			179	ND	179

^(a)^ND : NonDetect, TEM analytical sensitivity : (448 −896 s/cm^2^). ^(b)^One walking activity wipe sample (not reported) revealed chrysotile asbestos, not amphibole asbestos.

**Table 4:** Postactivity TEM chainsaw bar wipe sample results reported as concentration of amphibole asbestos <5 microns long, >5 microns long, and total structures per square centimeter.

Activity Performed	TEM (s/cm^2^) <5 *μ*m	TEM (s/cm^2^) >5 *μ*m	TEM (s/cm^2^) Total Asbestos
Fire Line Construction	8,600	3,225	11,825
Trail Maintenance	2,688	ND^(a)^	2,688
Fire Line Construction	896	ND^(a)^	896

^(a)^ND : NonDetect; TEM analytical sensitivity : (896 s/cm^2^).

## Abbreviations

AHERA: Asbestos Hazard Emergency Response Act
AIHA: American Industrial Hygiene Association
AS: Analytical Sensitivity
ASTM: American Society for Testing and Materials
ATSDR: Agency for Toxic Substances and Disease Registry
Ca: Calcium
COBRE: Center of Biomedical Research Excellence
EDS: Energy Dispersive X-ray Spectroscopy
EPA: Environmental Protection Agency
Fe: Iron
FS: Forest Service
GPS: Global Positioning System
K: Potassium
LOD: Limit of Detection
Mg: Magnesium
Na: Sodium
NIH: National Institute of Health
NIOSH: National Institute for Occupational Safety and Health
NMAM: NIOSH Manual of Analytical Methods
NVLAP: National Voluntary Laboratory Accreditation Program
OSHA: Occupational Safety and Health Administration
PBZ: Personal Breathing Zone
PCM: Phase Contrast Microscopy
PPE: Personal Protective Equipment
SD: Standard Deviation
SEM: Scanning Electron Microscope
TEM: Transmission Electron Microscopy
TWA: Time Weighted Average
USDA: United States Department of Agriculture.
